# A comprehensive review on the silane-acid reduction of alkenes in organic synthesis†

**DOI:** 10.1039/d5ra07101a

**Published:** 2025-11-21

**Authors:** Bapurao B. Shingate

**Affiliations:** a Department of Chemistry, Dr Babasaheb Ambedkar Marathwada University Chhatrapati Sambhajinagar 431 004 Maharashtra India bbshingate_chem@bamu.ac.in bapushingate@gmail.com

## Abstract

Target and diversity-oriented synthesis represents a versatile and efficient strategy for constructing structurally complex and privileged scaffolds from readily or commercially accessible starting materials. The combination of reagents indeed plays a pivotal role in organic synthesis, acting as chemical “tools” that enable specific reactions to occur and driving the creation of new molecules. Reagents facilitate organic transformations, including the controlling of reaction pathways and influencing the complex efficiency and selectivity of the synthesis process. This review highlights the combined use of triethylsilane and trifluoroacetic acid as a powerful system for the chemoselective and regioselective ionic hydrogenation of diverse alkenes. The transformation proceeds through protonation, followed by hydride transfer, affording valuable products with high selectivity. Furthermore, this review covers the reduction of heterocyclic skeletons to saturated compounds *via* the ionic hydrogenation method.

## Abstract 2

Target and diversity-oriented synthesis represents a versatile and efficient strategy for constructing structurally complex and privileged scaffolds from readily or commercially accessible starting materials.

## Graphical abstract

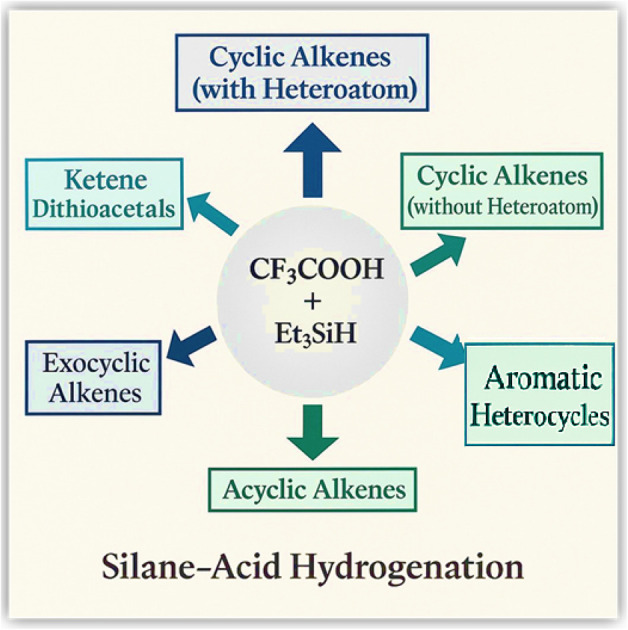

## 1. Introduction

Organic synthesis has enormously contributed to improving the living standards and life expectancy of society by providing value-added materials like pharmaceuticals, polymers, textiles, dyes, agrochemicals and smart materials required for electronic device applications. Organic synthesis is considered a constructive science and has played a pivotal role in developing countless number of non-natural molecules. Organic synthesis includes the development of carbon–carbon bond(s) and carbon–heteroatom bond(s) and cleavage of these bonds.^1–5^

The construction and cleavage of bonds using various strategies represent the central idea in organic chemistry, playing an excellent role in assembling the complex carbon frameworks. Thus, the development of different approaches has remained the main focus of synthetic organic chemistry research. The development of carbon–carbon bond is the most essential reaction due to its unique role in the formation of various classes of carbon frameworks.^6–8^ There are several significant carbon–carbon bond-forming reactions/rearrangements, and organometallic reagents have been developed and studied in detail for their applications during the current and last centuries. Furthermore, organic functional group transformations, such as oxidation and reduction, are the key steps in the synthesis of natural products, drugs and complex molecules.^9–11^

Hydrogenation has become a significant process in synthetic organic chemistry.^12,13^ The successful synthesis of many new compounds often relies on the ability to achieve the selective reduction of a single unsaturated group within a molecule while leaving other functionalities unaffected. The selection of an appropriate hydrogenating system for targeted hydrogenation requires an understanding of the mechanism by which this system operates, and this selection relies on the behavior of the unsaturated group that interacts with the hydrogenating system.

In organic synthesis, reagents play a vital role in facilitating chemical transformations and enabling the conversion of starting materials into the desired products. They can be classified according to their functions, such as oxidizing agents, reducing agents, or those employed in specific named reactions.^14–17^ Trifluoroacetic acid (TFA) is widely used in organic synthesis as a catalyst, reagent and solvent. Several synthetic organic transformations, including rearrangements, condensations, oxidations, reductions, hydroarylations, trifluoromethylations, and functional group deprotections, have been performed using trifluoroacetic acid.^18,19^

Organosilanes interact with various unsaturated carbon–carbon and carbon–heteroatom bonds through the addition reaction of hydrogen and silicon atoms, most probably in hydrosilylation, and they have been employed in organic synthesis.^20^ The Si–H bond exhibits lower ionic character and shows stability in the presence of water; therefore, hydrosilylation reactions are conducted using transition metal catalysts.^21^ These compounds are comparatively less toxic, making their use potentially environmentally benign.

Recent developments have highlighted the use of sustainable and bench-stable reductants, particularly polymethylhydrosiloxane (PMHS), which offers practical and environmentally benign alternatives to conventional hydrosilanes. PMHS has been extensively utilized as a mild and efficient reducing agent in a wide range of functional group transformations, highlighting its importance in modern synthetic chemistry.^22–24^

The scope of silane reductions has further expanded through enantioselective hydrosilylation, where chiral metal complexes enable asymmetric reductions of carbonyl and imine substrates to yield optically active alcohols and amines.^25^ Furthermore, several reports have demonstrated the versatility of silanes in the reduction of diverse functional groups.^26–28^ Over the past decades, a wide range of transition metal catalysts, based on platinum, rhodium, cobalt, iron, nickel, and copper, have been developed to mediate hydrosilylation and related silane reduction reactions of olefins with high activity and selectivity.^29,30^

Triethylsilane (TES) is a versatile reducing agent with broad applications across diverse substrates. Its unique properties highlight its significance in modern synthetic chemistry, particularly in chemo- and stereo-selective synthesis of complex molecular frameworks.^31–33^

**Scheme 1:**
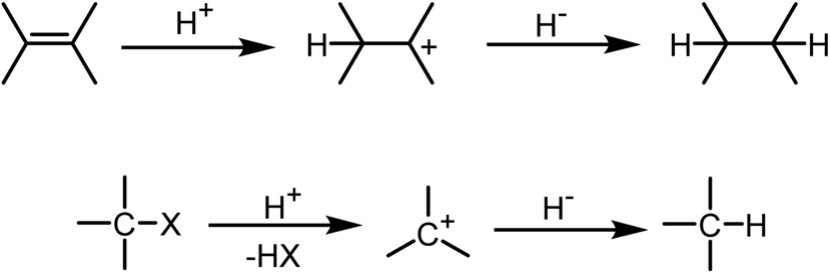
Mechanism of ionic hydrogenation.

Ionic hydrogenation relies on the ability of an unsaturated compound to undergo protonation, generating a reactive carbocation intermediate.^34–38^ The subsequent hydride transfer from a donor species to this carbocation affords the hydrogenated product. This strategy is applicable to the reduction of a wide range of functionalities, including carbon–carbon, carbon–oxygen, and carbon–nitrogen multiple bonds, as well as certain single bonds such as carbon–halogen and carbon–oxygen linkages. The basic principle of ionic hydrogenation involves the formation of a carbocation, either by protonation of a double bond or through heterolysis of a C–X bond, followed by its reduction *via* hydride donation to form the hydrogenation product (Scheme 1).

In ionic hydrogenation, the hydrogenating pair includes a proton donor and a hydride donor that must fulfill specific criteria: (a) the proton source should be sufficiently acidic to protonate the carbon–carbon double bond, forming a carbocation, but it should not be strongly acidic to protonate the hydride source and generate hydrogen. (b) The carbocation needs to be sufficiently electrophilic to capture a hydride from the hydride source and must not react with other nucleophiles present in the reaction system, such as the conjugate base of the proton source. The typical reduction system used for ionic hydrogenation of double bonds involves trifluoroacetic acid paired with an organosilane. Hydrosilanes have been utilized as mild reducing agents in fine organic synthesis.^39,40^

**Scheme 2:**
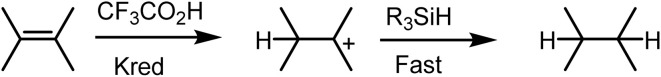
Rate-determining step.

The alkene substrate, however, must be susceptible to protonation by trifluoroacetic acid, which restricts the scope of this method primarily to the reduction of tri- and tetra-substituted alkenes as well as aryl-substituted alkenes (Scheme 2).

In ionic hydrogenation, the rate-determining step involves protonation of the double bond, followed by hydride transfer to the resulting carbocation. The efficiency of this process depends strongly on the nature and number of alkyl or aryl substituents attached to the silicon atom. The hydride-donating ability of silanes generally follows^41,42^ the order:Et_3_SiH > (*n*-C_8_H_19_)_3_SiH > Et_2_SiH_2_ > (C_6_H_5_)_2_SiH_2_ > (C_6_H_5_)_3_SiH > C_6_H_5_SiH_3_

The combination of triethylsilane and trifluoroacetic acid or Lewis acids is used for reduction reactions, such as carbonyls to alcohols,^43,44^ carbonyls to alkanes,^45,46^ allylic/benzylic/tertiary/propargylic alcohols to alkanes,^47–51^ hemiaminals to hydrocarbons,^52^ lactols/hemiacetals^53,54^ to hydrocarbons and many more.^55–62^ Furthermore, triethylsilane and trifluoroacetic acid or Lewis acids are employed for the reductive cleavage of spiroketals,^63^ benzylidene acetals,^64^ oxazolidinones,^65^ bicyclic lactams^66^ and the reduction of imines^67^ and aromatic nitro^68^ functionalities.

The potential of ionic hydrogenation reaction, its unique characteristics, and a comprehensive review of the application of silane-acid reductions to different types of alkenes have not been thoroughly covered in previous literature. This review presents the combination of trifluoroacetic acid and triethylsilane for the reduction of acyclic alkenes, ketene dithioacetal, exocyclic double bonds, cyclic double bonds with and without heteroatoms and aromatic heterocycles.

## 2. Silane-acid reduction of alkenes

Alkenes amenable to ionic hydrogenation are those capable of generating stabilized carbocations, such as branched alkenes, alkylcyclopropenes, and substituted styrenes. In contrast, unbranched alkenes or those branched at positions other than the alkenic carbon generally do not undergo reduction. This method, therefore, enables the selective hydrogenation of highly substituted double bonds even in the presence of an unsubstituted one. This regioselectivity is opposite to that typically observed in catalytic hydrogenation.

### 2.1 Reduction of acyclic alkenes

**Scheme 3:**
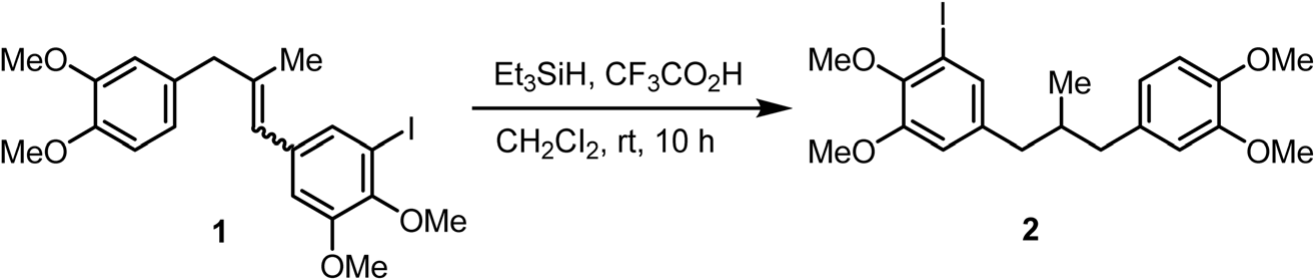
Ionic hydrogenation of alkenes.

Olefinic compounds bearing bromo- or iodo-substituents are often susceptible to dehalogenation under catalytic hydrogenation conditions. In contrast, ionic hydrogenation does not typically affect such functionalities. Kramer and Waldvogel demonstrated^69^ the selective ionic hydrogenation of an iodo-substituted substrate 1 with triethylsilane (Et_3_SiH) and trifluoroacetic acid (CF_3_CO_2_H) in dichloromethane and obtained the saturated compound 2 in quantitative yield (Scheme 3).

**Scheme 4:**
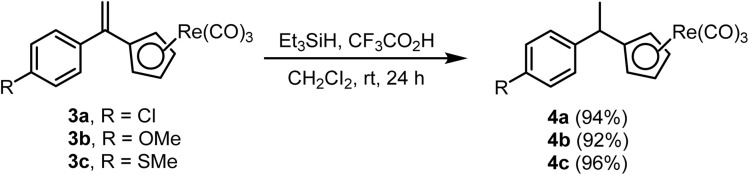
Reduction of the double bond in an organometallic compound.

The ionic hydrogenation method is also used for the reduction of double bonds in organometallic compounds. A series of compounds 4 was synthesized^70^ from 3 using Et_3_SiH and CF_3_CO_2_H in nearly quantitative yields (Scheme 4).

**Scheme 5:**
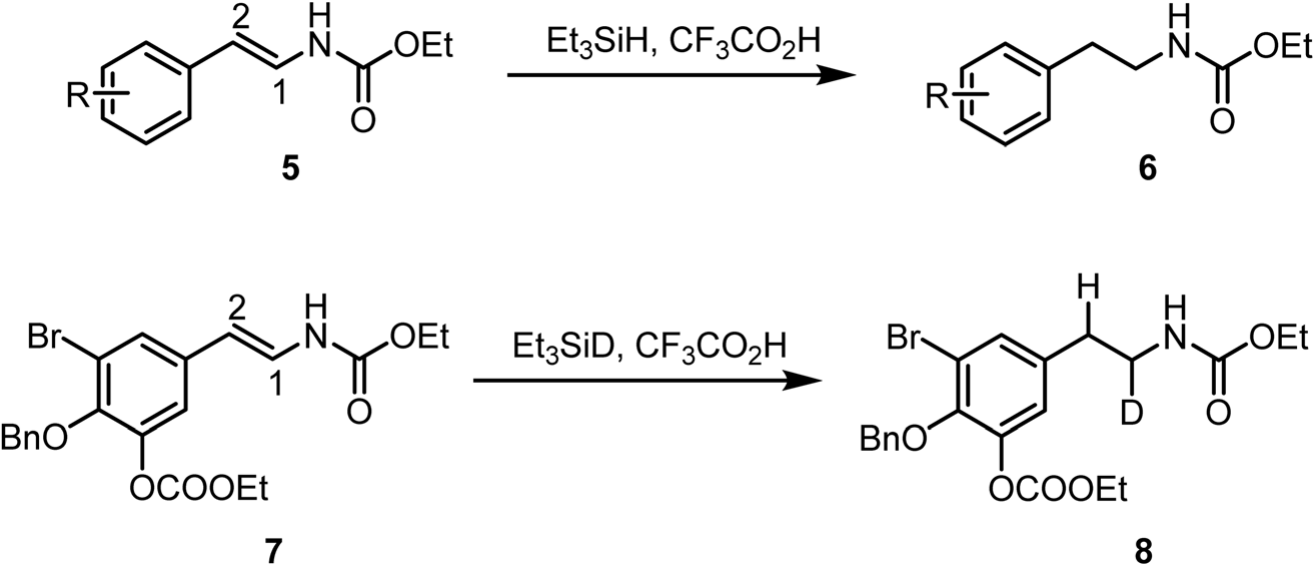
Reduction of *N*-carboalkoxyenamines using ionic hydrogenation.

Masuno and Molinski have reported^71^ the selective reduction of 2-aryl-1-*N*-carboalkoxyenamines 5 to the corresponding 2-arylethylamine carbamates 6 by using Et_3_SiH in the presence of CF_3_CO_2_H in excellent yields. The reaction proceeds *via* hydride addition at the C-1 position, with the rate-determining step involving proton transfer from CF_3_CO_2_H. The mechanism was further investigated by comparing the reaction rates with deuterated *versus* non-deuterated reagents. When Et_3_SiD was employed instead of Et_3_SiH for the reduction of compound 7, efficient conversion to deuterium-labeled arylethylamine 8 was observed within a similar reaction time (Scheme 5).

**Scheme 6:**
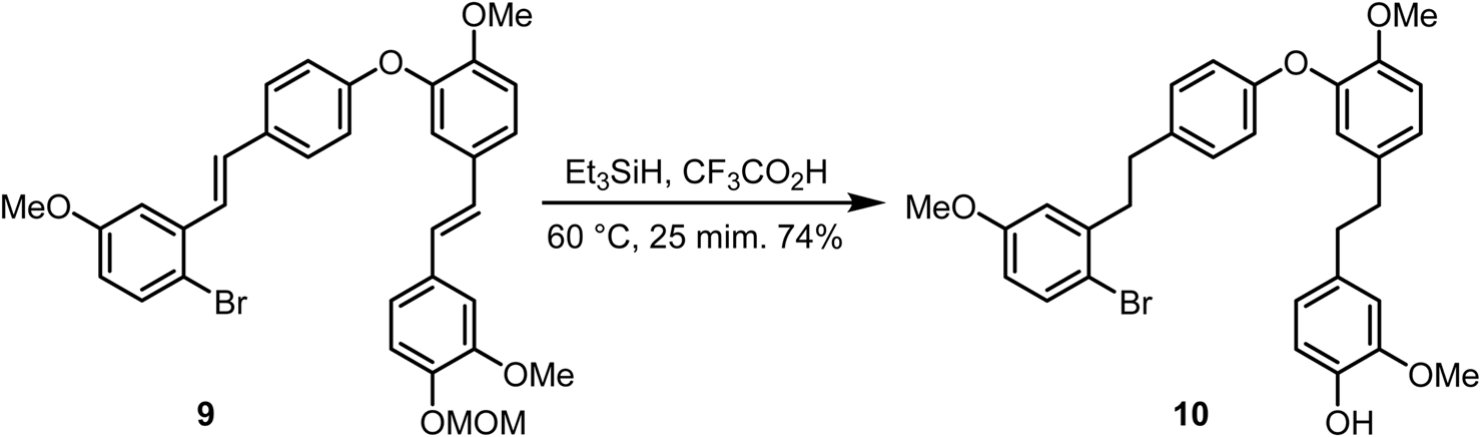
Reduction of double bonds and cleavage of the MOM group.

Hioki and co-workers reported the reduction^72^ of double bonds in compound 9 using triethylsilane in trifluoroacetic acid at 60 °C to compound 10, in which reductive cleavage of the MOM group also occurs (Scheme 6).

**Scheme 7:**
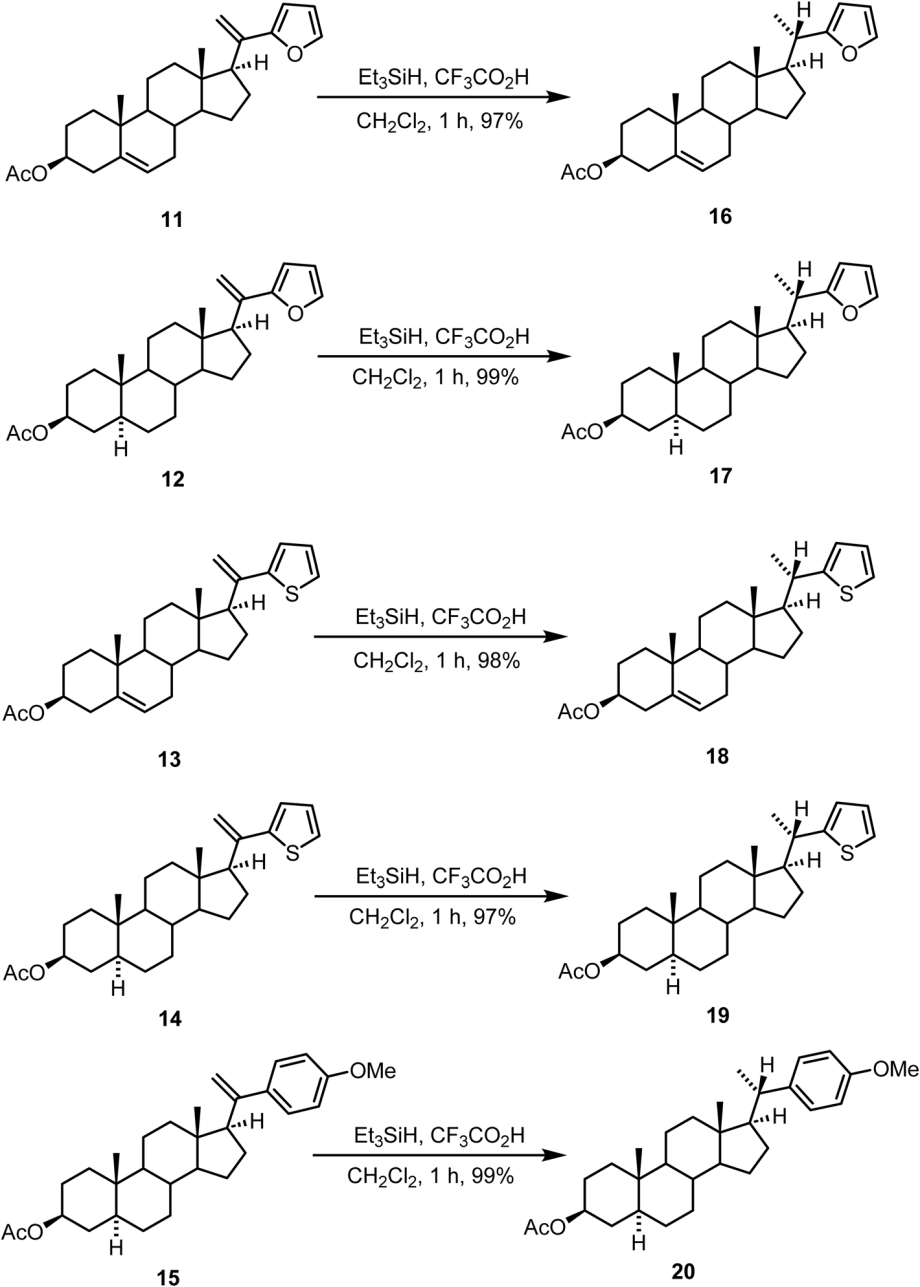
Stereoselective ionic hydrogenation of steroidal C-20(21)-olefinic double bonds.

The stereoselective ionic hydrogenation of steroidal C-20(21)-olefinic double bond was achieved in excellent yields.^73^ Ionic hydrogenation of the steroidal C-20(21)-olefinic double bond in compounds 11–15 with Et_3_SiH and CF_3_CO_2_H in CH_2_Cl_2_ at 30 °C resulted in the corresponding 16–20 in almost quantitative yields (Scheme 7). Ionic hydrogenation of compounds 11 and 13 is chemoselective as the 5,6-double bond is unaffected.

**Scheme 8:**
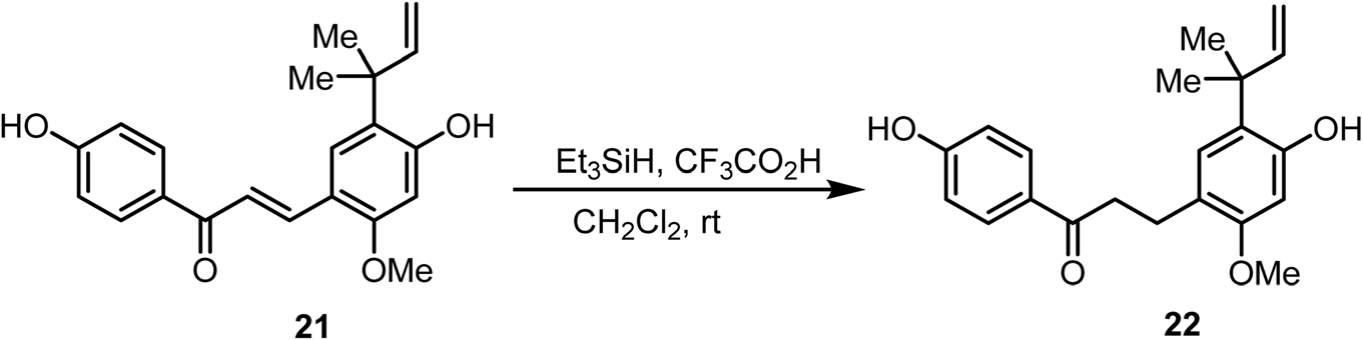
Reduction of the chalcone double bond.

Selective reduction of the chalcone double bond (α,β-unsaturated) in compound 21 was achieved^74^ by ionic hydrogenation using trifluoroacetic acid as the proton donor and triethylsilane as the hydride donor. The side-chain double bond, being poorly polarized, remained unreactive under these conditions. Furthermore, employing equimolar concentrations of silane and chalcone prevented the reduction of the carbonyl group. The reaction afforded saturated ketone 22 in high yields, which was readily isolated from the mixture (Scheme 8).

#### 2.1.1 Reduction of ketene dithioacetals

**Scheme 9:**
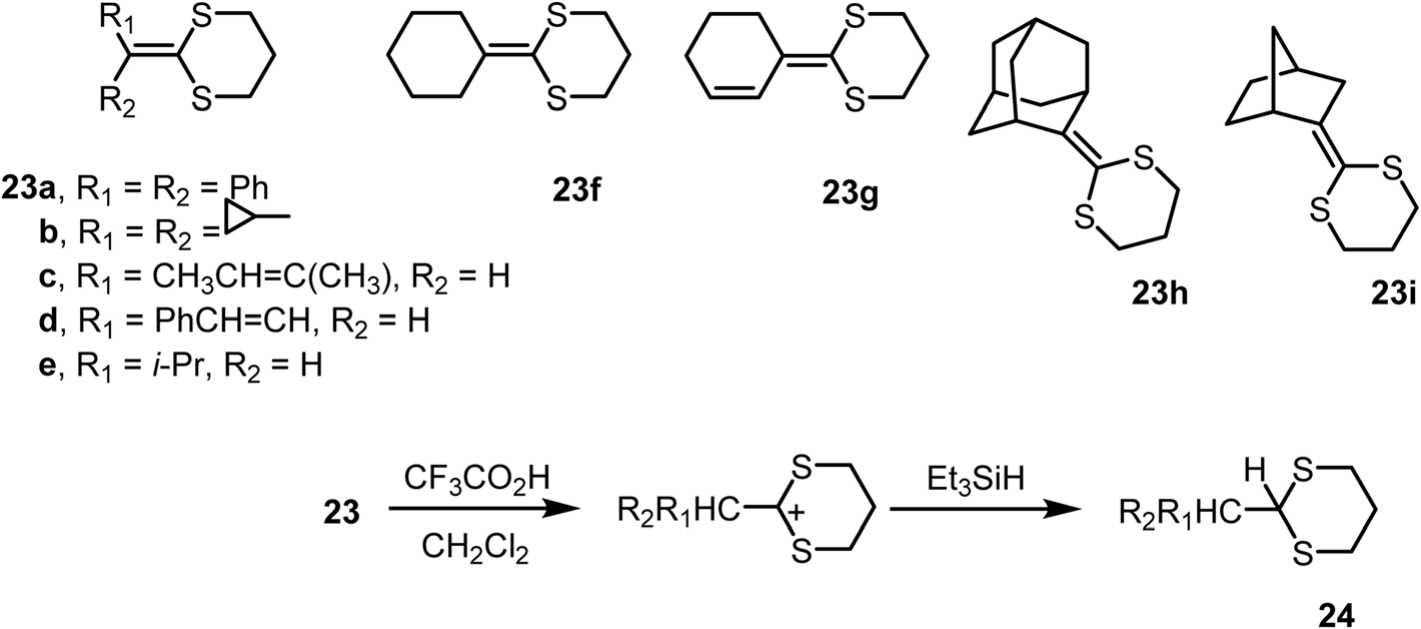
Ionic hydrogenation of ketene thioacetals.

Several ketene thioacetals were reduced^75^ to thioacetals *via* a protonation-hydride transfer sequence using Et_3_SiH and CF_3_CO_2_H in dichloromethane, demonstrating the utility of this reaction for converting R^1^R^2^C=C=O into R^1^R^2^CHCHO. Evidence indicated that stabilization of the adjacent carbocation through electron donation from sulfur played a significant role in the process. Ketene thioacetals were generated by the metalation of 2-trimethylsilyl-1,3-dithiane with n-butyllithium in THF, followed by the reaction with aldehydes and ketones. Benzophenone was converted into diphenylacetaldehyde by the reduction of 23a (R^1^=R^2^=Ph) to 24, followed by oxidative hydrolysis of 24 to Ph_2_CHCHO. The reduction step, performed with Et_3_SiH and CF_3_CO_2_H in dichloromethane, proceeded in 87% yield (Scheme 9).

**Scheme 10:**
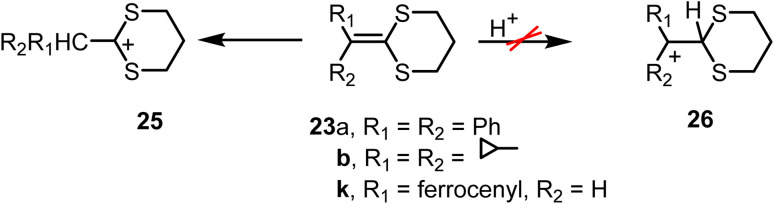
Protonation of ketene dithioacetals.

Compound 23f was prepared from cyclohexanone and reduced to 2-cyclohexyl-1,3-dithiane in 63% yield, and on hydrolysis, furnished cyclohexanecarboxaldehyde in 93% yield.^75^ The diphenyl and dicyclopropyl ketene thioacetals (23a and 23b) were particularly useful in probing the site of protonation in ketene thioacetals. For the ferrocene-derived ketene thioacetal 23k, evidence indicated that protonation occurs at the carbon atom adjacent to the ferrocene moiety, generating the sulfur-stabilized carbocation 25, rather than at the dithiane ring to form the ferrocenylmethyl cation 26. This observation highlights the strong stabilizing effect of sulfur, most likely through electron donation from its lone pairs to the adjacent carbocation. This is notable because ferrocenylmethyl cations are themselves recognized as highly stable ions (Scheme 10).

**Scheme 11:**
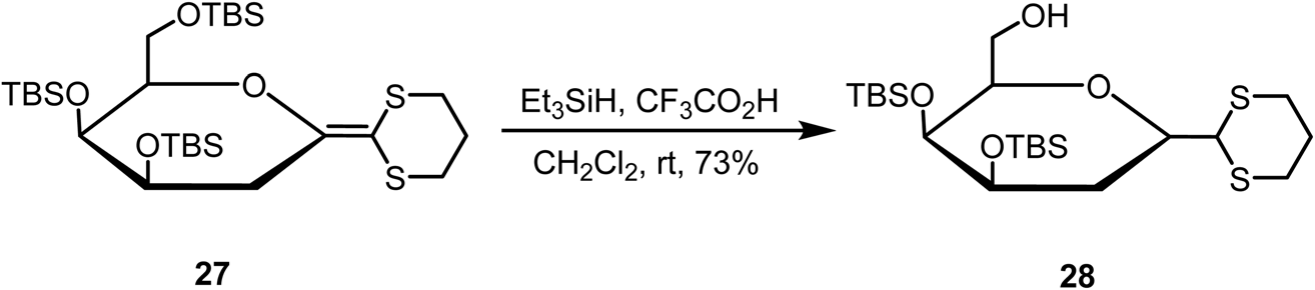
Reduction of ketene dithioacetal.

Mlynarski and Banaszek reported^76^ the reduction of the double bond of ketene dithioacetal 27 with Et_3_SiH and CF_3_CO_2_H in dichloromethane at room temperature, affording the saturated compound 28 in 73% yield (Scheme 11). The primary silyl ether group of 21 is also selectively cleaved under these conditions.

**Scheme 12:**
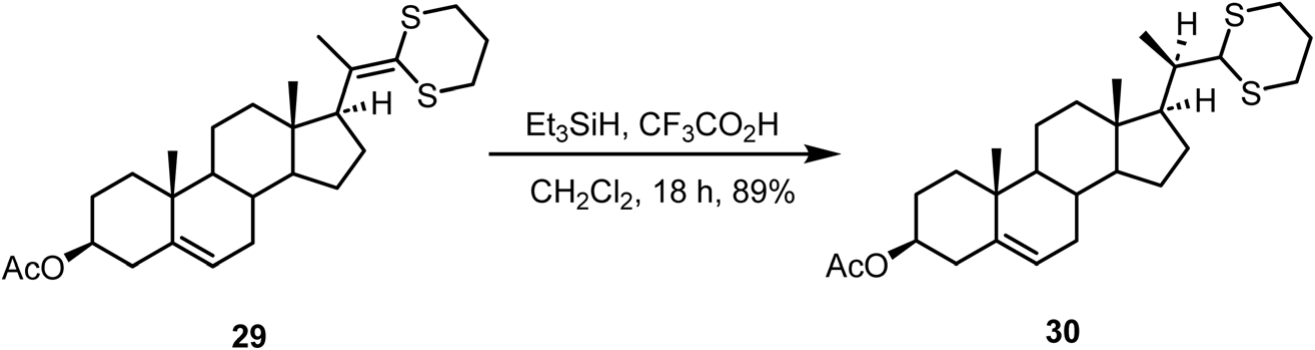
Ionic hydrogenation of the steroidal C-20,22-ketene dithioacetal.

Ionic hydrogenation of the steroidal C-20,22-ketene dithioacetal 29, prepared from commercially available^77^ 16-dehydropregnenolone acetate with triethylsilane and trifluoroacetic acid in dichloromethane at 25 °C for 18 h afforded^78,79^ the C(20R) saturated compound 30 in 89% yield (Scheme 12).

#### 2.1.2 Reduction of exocyclic double bonds

**Scheme 13:**
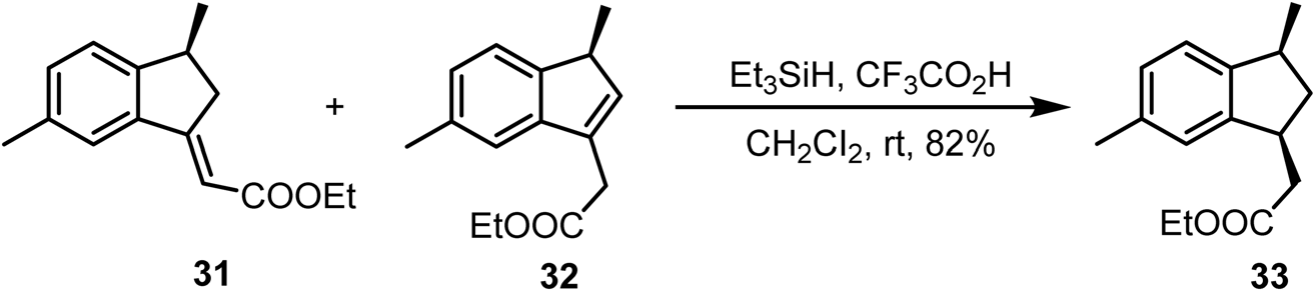
Reduction of unsaturated ester.

Ho and co-workers reported^80^ the reduction of double bonds from the mixture of unsaturated esters 31 and 32 using Et_3_SiH and CF_3_CO_2_H in CH_2_Cl_2_ at room temperature, which afforded the saturated ester 33 in 82% yield (Scheme 13). Notably, the ester functionality remained unaffected under these conditions.

**Scheme 14:**
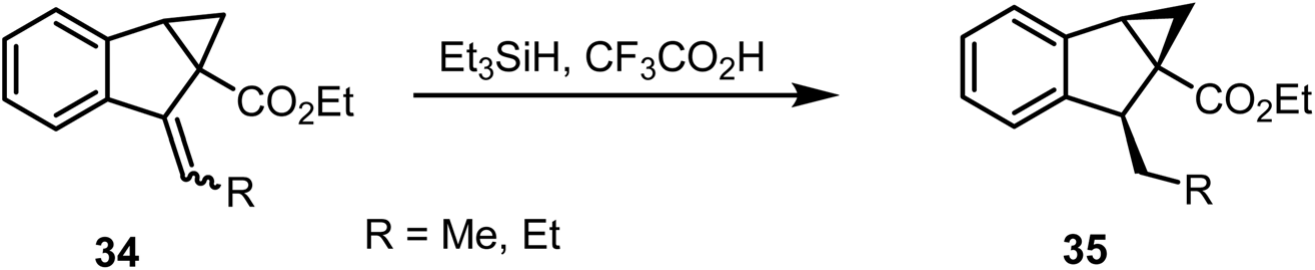
Reduction of exocyclic olefins.

Vacher and co-workers reported^81^ the chemoselective reduction of an *exo*-olefin in ester 34 with triethylsilane and trifluoroacetic acid, affording ester 35 in good yields (Scheme 14). Interestingly, the cyclopropane ring remained unaffected under these conditions.

**Scheme 15:**
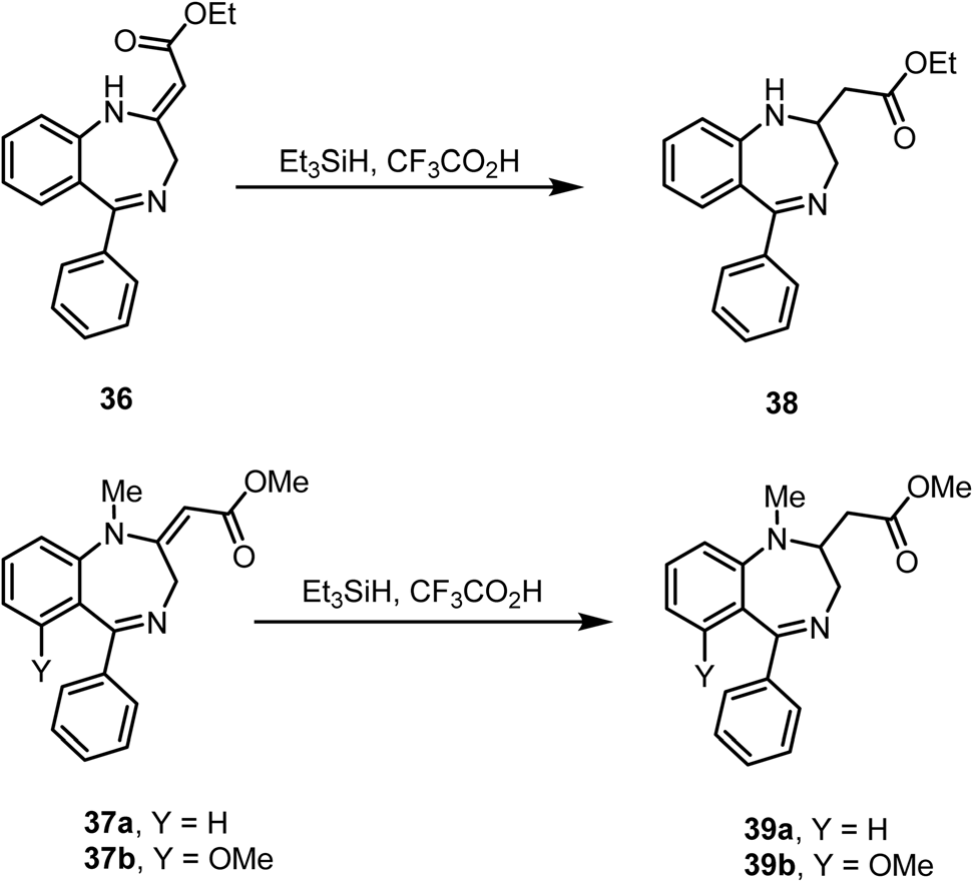
Reduction of an exocyclic unsaturated ester.

Anzini and co-workers reported^82^ the chemoselective reduction of double bonds in unsaturated esters 36, 37a and 37b with triethylsilane in trifluoroacetic acid, providing the corresponding saturated esters 38, 39a and 39b, respectively, in good yields (Scheme 15).

**Scheme 16:**
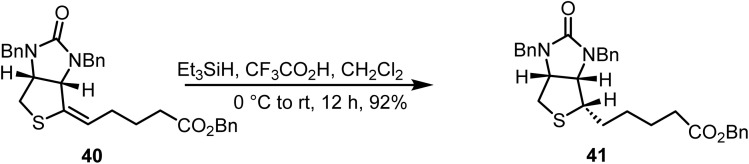
Ionic hydrogenation of an exocyclic double bond.

Huang and co-workers reported^83^ the selective reduction of a double bond in compound 40 using triethylsilane and trifluoroacetic acid in dichloromethane, affording compound 41 in 92% yield (Scheme 16).

**Scheme 17:**
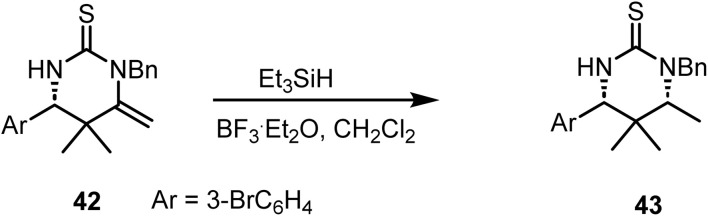
Reduction of an exocyclic double bond.

Li and co-workers reported^84^ the diastereoselective reduction of dihydropyrimidine thione 42 with triethylsilane and BF_3_·Et_2_O in dichloromethane to chiral thiourea 43 in 81% yield (Scheme 17).

### 2.2 Reduction of cyclic double bonds

#### 2.2.1 Reduction of cyclic double bonds without a heteroatom in the ring

**Scheme 18:**
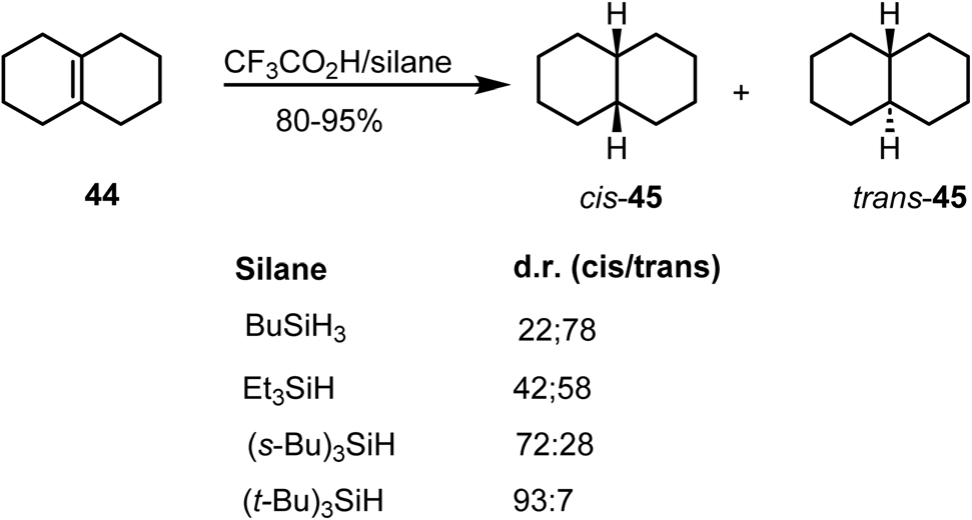
Ionic hydrogenation of Δ^9(10)^-octalin.

The stereoselectivity of double-bond reduction in ionic hydrogenation is governed by steric accessibility and is highly sensitive to both the substrate structure and the choice of hydride donor.^85^ The ionic hydrogenation of olefin 44 (Scheme 18) illustrates that the steric size of the hydride source plays a decisive role. The ionic hydrogenation of Δ^9(10)^-octalin with CF_3_CO_2_H and various silanes demonstrated pronounced stereoselectivity. When BuSiH_3_ was employed as the hydride donor, the reaction furnished *cis*- and *trans*-decalin 45 in a 22 : 78 ratio. In contrast, the use of bulky ^*t*^Bu_3_SiH predominantly afforded the opposite stereoisomer, yielding 93% of the *cis*-decahydronaphthalene product.

**Scheme 19:**
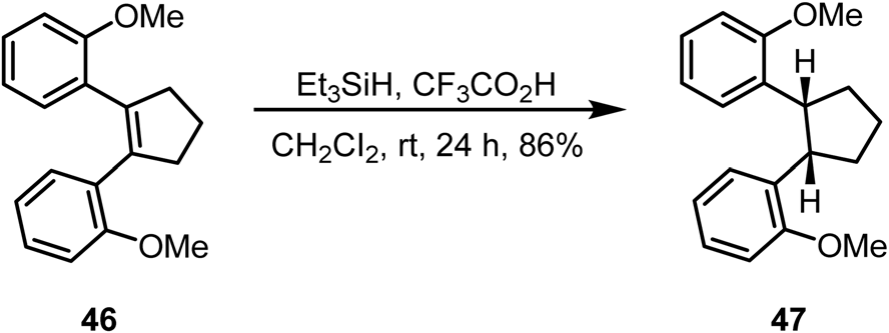
Ionic hydrogenation of *tetra*-substituted cyclopentene.

The effect of substituents on olefins is important in the reduction reaction. Whiteseil and Apodaca reported^86^ a *tetra*-substituted cyclopentene derivative 46 on ionic hydrogenation with Et_3_SiH and CF_3_CO_2_H in dichloromethane to *cis*-cyclopentane 47 in 86% yield (Scheme 19).

**Scheme 20:**
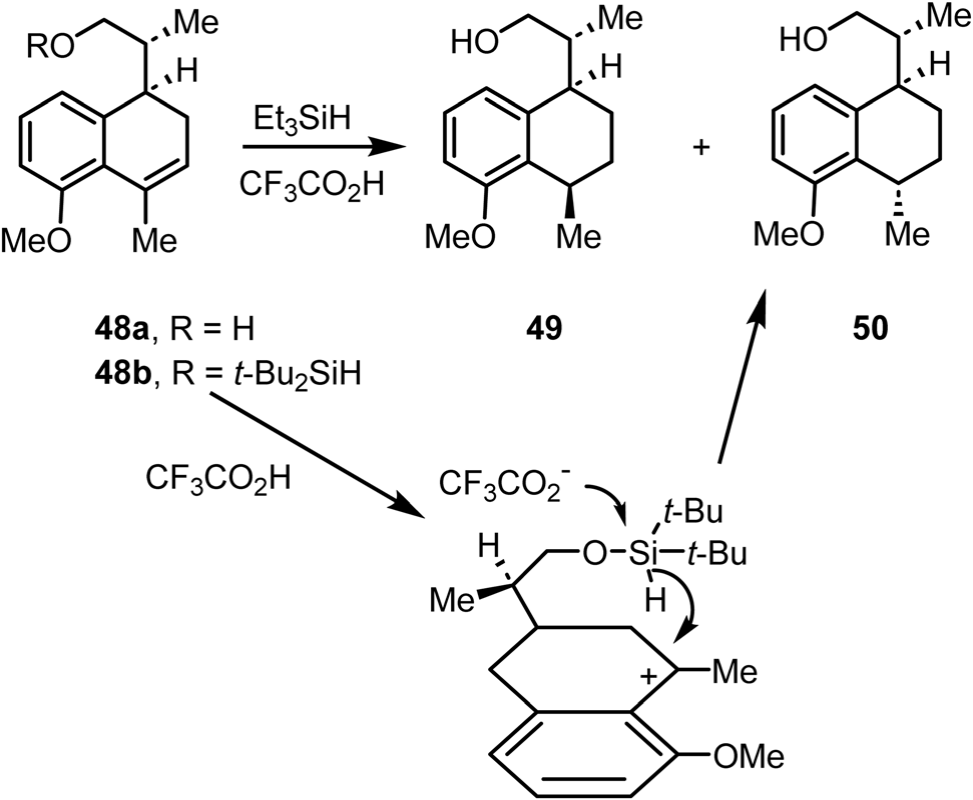
Ionic hydrogenation of compound 48.

McCombie and co-workers reported^87^ the ionic hydrogenation of compound 48 with Et_3_SiH and CF_3_CO_2_H to a 2 : 3 mixture of 49 and 50 (Scheme 20). Furthermore, the intramolecular variant of this methodology was shown to effectively control the stereochemical outcome. The silyl ether 48b on reaction with trifluoroacetic acid in dichloromethane yielded compound 50 with >95% enantiomeric purity (Scheme 20).

**Scheme 21:**
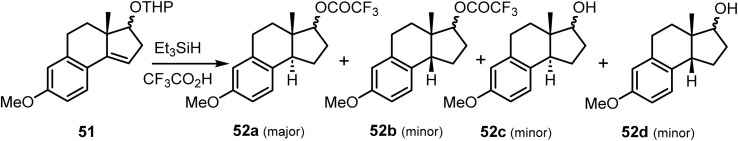
Ionic hydrogenation of compound 51.

Ravindranathan and co-workers reported^88^ the ionic hydrogenation of compound 51 with Et_3_SiH and CF_3_CO_2_H at 0 °C, which afforded the isomeric mixture of trifluoroacetates 52a and 52b (44%, 2 : 1), along with alcohols 52c and 52d (28%, 2 : 1) (Scheme 21).

**Scheme 22:**
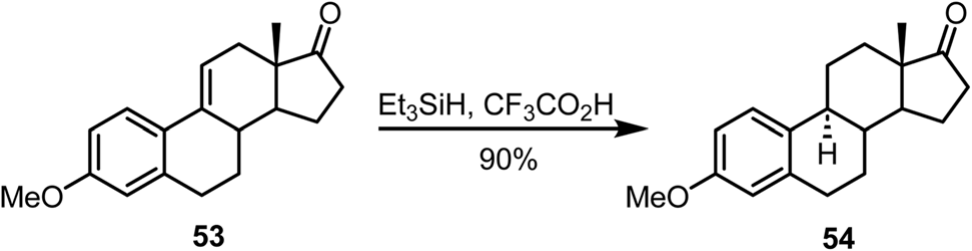
Ionic hydrogenation of Δ^9(11)^-estrone derivative.

Posner and Switzer reported^89^ the synthesis of estrone methyl ether with exceptionally high enantiomeric purity by ionic hydrogenation of Δ^9(11)^-estrone derivative 53 using Et_3_SiH and CF_3_CO_2_H, which afforded compound 54 in 90% yield (Scheme 22).

**Scheme 23:**
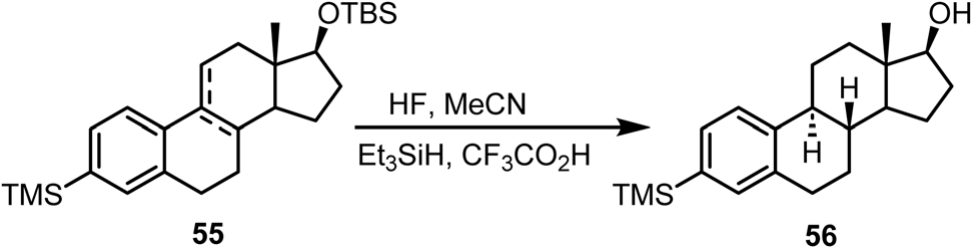
Ionic hydrogenation of compound 55.

The mixture of compound 55 was first treated with HF/MeCN to remove the hydroxyl protecting group, and the resultant alcohols were subsequently subjected^90^ to Et_3_SiH and CF_3_CO_2_H in benzene, which provided the desired *trans*-fused tetracycle 56 in 45% yield (Scheme 23).

**Scheme 24:**
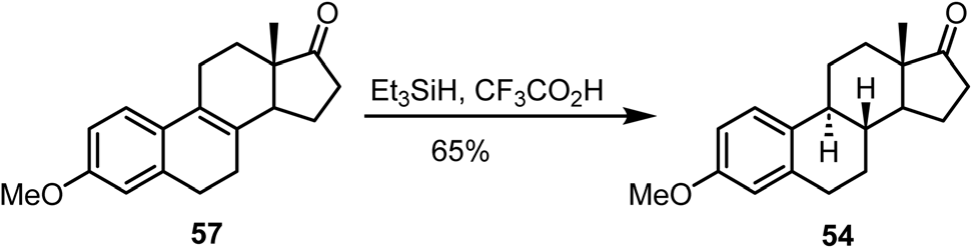
Ionic hydrogenation of Δ^8(9)^-estrone.

Sugahara and Ogasawara reported^91^ the ionic hydrogenation of Δ^8(9)^-estrone derivative 57 with triethylsilane and trifluoroacetic acid, which afforded compound 54 in 65% yield (Scheme 24).

**Scheme 25:**
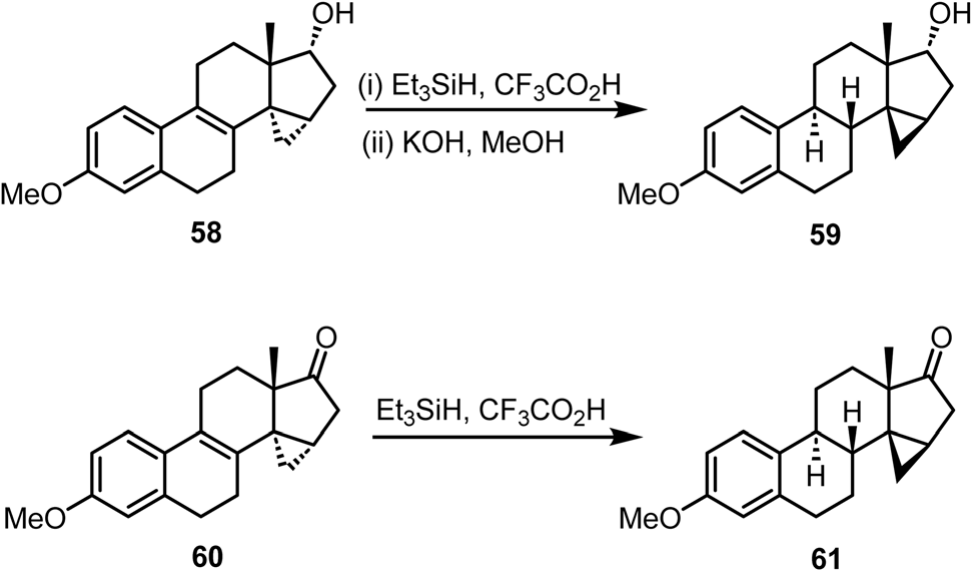
Ionic hydrogenation of Δ^8(9)^-estrone derivatives.

Schwarz and coworkers reported^92^ the ionic hydrogenation of 3-methoxy-14α,15α-methylenestra-1,3,5(10),8-tetraen-17α-ol 58 with triethylsilane and trifluoroacetic acid, resulting in 3-methoxy-14β,15β-methylenestra-1,3,5(l0)-trien-17α-ol 59, rather than the 14α,15α-methylene-9β product. Furthermore, ionic hydrogenation of the 8-double bond in compound 60 predominantly yielded an 8β,9α-dihydro product. However, this ionic hydrogenation process was accompanied by an additional inversion of the 14α,15α-methylene bridge to compound 61 (Scheme 25).

**Scheme 26:**
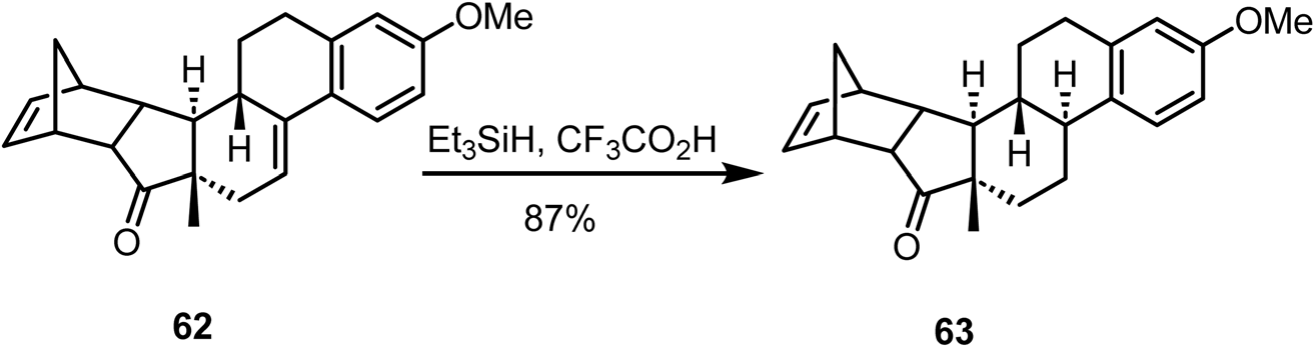
Chemoselective ionic hydrogenation of 62.

Takano and co-workers reported^93^ the chemoselective ionic hydrogenation of 62 using triethylsilane and trifluoroacetic acid, which afforded the *trans*-B/C fused product 63 in 87% yield (Scheme 26). This intermediate 63 was converted to (+) estrone *via* a multistep synthesis.

**Scheme 27:**
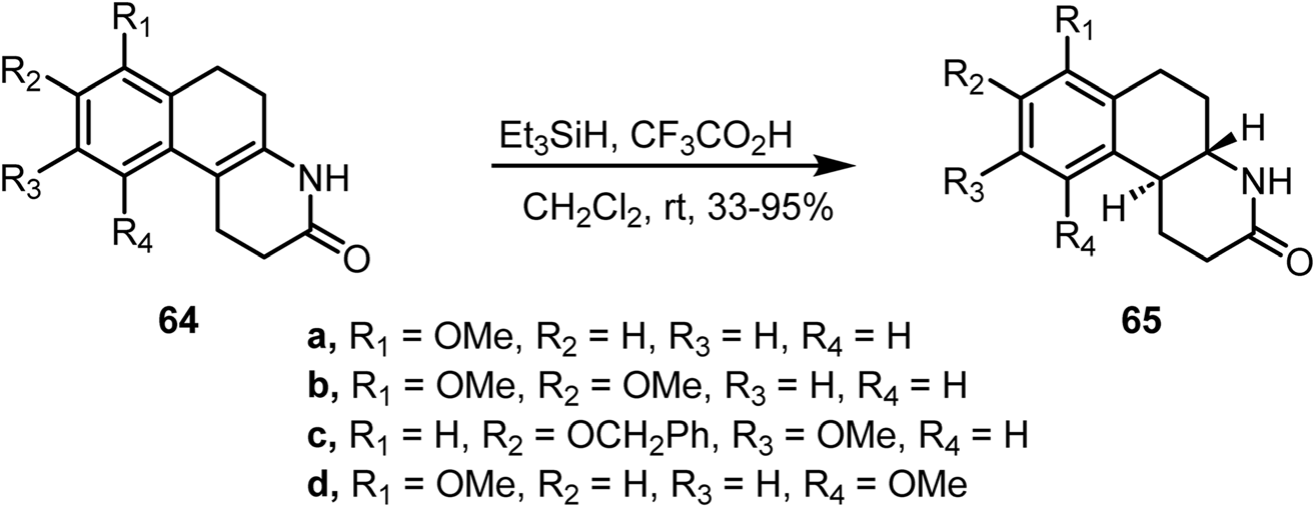
Reduction of a fused double bond.

Cannon and co-workers reported^94^ the reduction of a fused carbocyclic ring system containing a carbon–carbon double bond shared by two rings. The ionic hydrogenation with trifluoroacetic acid and triethylsilane in dichloromethane at room temperature yielded the *trans*-fused ring fusion. In this study, application of this hydrogenation method to a series of tetrahydroquinolines 64 provided the corresponding *trans*-fused lactams 65 in 33–95% yield (Scheme 27).

**Scheme 28:**
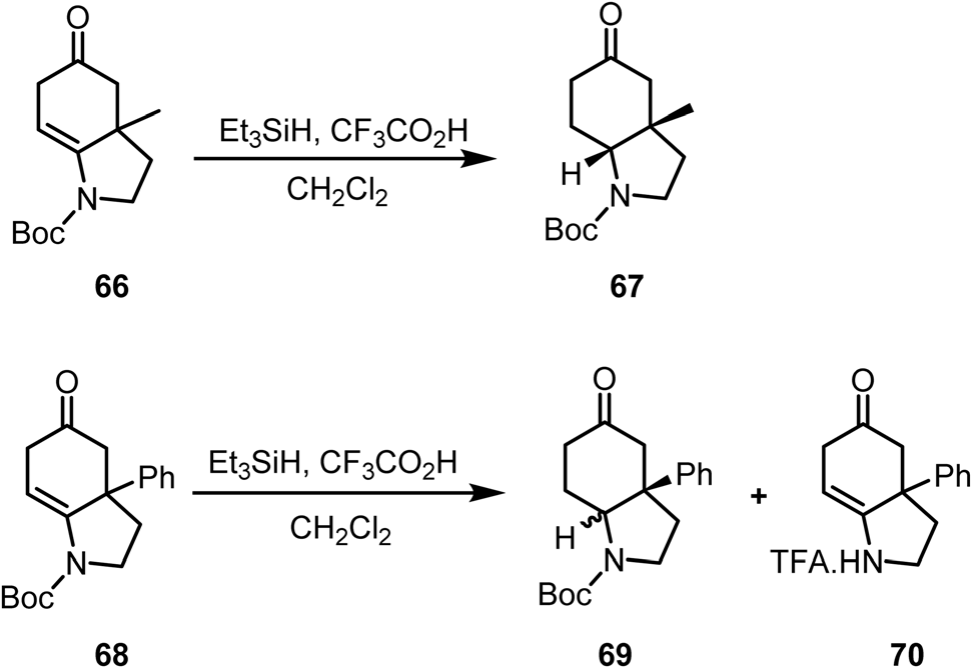
Reduction of enamines by ionic hydrogenation.

Under ionic reduction conditions (triethylsilane/trifluoroacetic acid), the enamine group of methyl-*N*-Boc-hexahydro-1*H*-indolin-5(6*H*)-one 66 was reduced^95^ to afford exclusively a *cis*-fused product 67. In contrast, the reduction of phenyl-*N*-Boc-hexahydro-1*H*-indolin-5(6*H*)-one 68 furnished a distereomeric mixture 69, along with a minor amount of the Boc-deprotected compound 70 (Scheme 28).

**Scheme 29:**
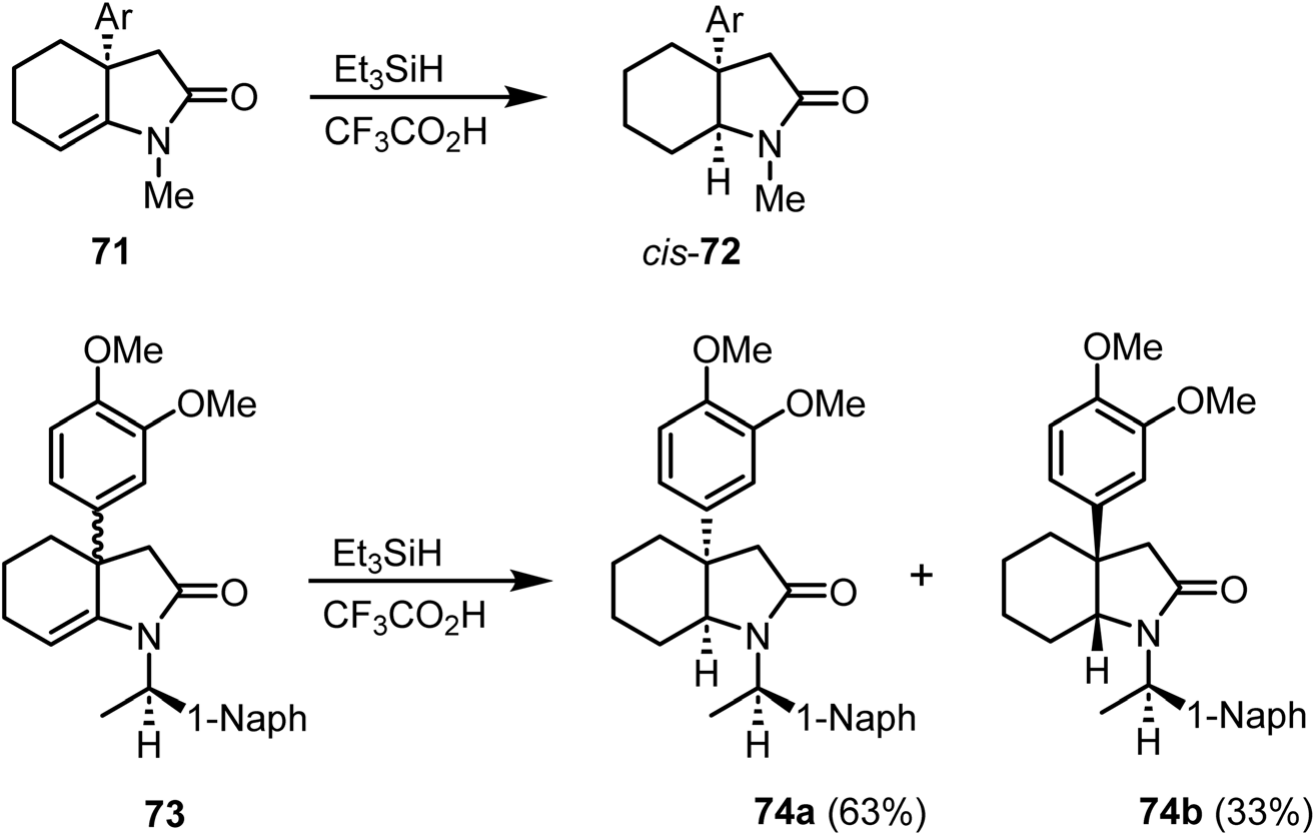
Reduction of a fused double bond by ionic hydrogenation.

Saito *et al.* reported^96^ the ionic hydrogenation of compound 71 with triethylsilane and trifluoroacetic acid, which furnished the *cis*-fused aryloctahydroindol-2-one 72 as the sole product (Scheme 28). Similarly, reduction of the distereomeric mixture of hexahydroindol-3-ones 73 under similar conditions afforded octahydroindol-2-one 74a as the major product and its isomer 74b as a minor product (Scheme 29).

#### 2.2.2 Reduction of cyclic double bonds with a heteroatom in the ring

**Scheme 30:**
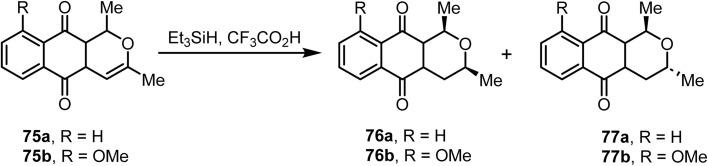
Ionic hydrogenation of a double bond.

The reduction^97,98^ of naphthopyrandione 75b with triethylsilane and trifluoroacetic acid afforded eleutherin 76b and isoeleutherin 77 (Scheme 30). Eleutherin 76b and isoeleutherin 77 are antibiotics found in *Eleutherine bulbosa*. Similarly, the reduction of compound 75a under identical reaction conditions at room temperature afforded *cis*-1,3-dimethyl-3,4-dihydro-1*H*-naphtho[2,3-*c*]pyran-5,10-dione 76a in excellent yield. Furthermore, compound 75b under similar reaction conditions resulted in a 1 : 5 mixture of 76b and its diastereoisomer (±)-isoeleutherin 77 in moderate yields (Scheme 30). In these transformations, the hydride source from triethylsilane determines the stereochemical outcome of the products.

**Scheme 31:**
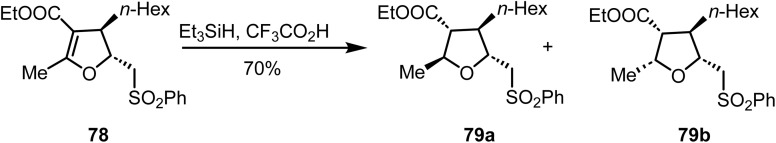
. Reduction of dihydrofuran derivative 78.

The highly substituted dihydrofuran derivative 78 on ionic hydrogenation^99^ with Et_3_SiH in CF_3_CO_2_H at 60 °C afforded an 86 : 14 mixture of enantiomerically pure tetrahydrofurans 79a and 79b in 70% yield (Scheme 31).

**Scheme 32:**
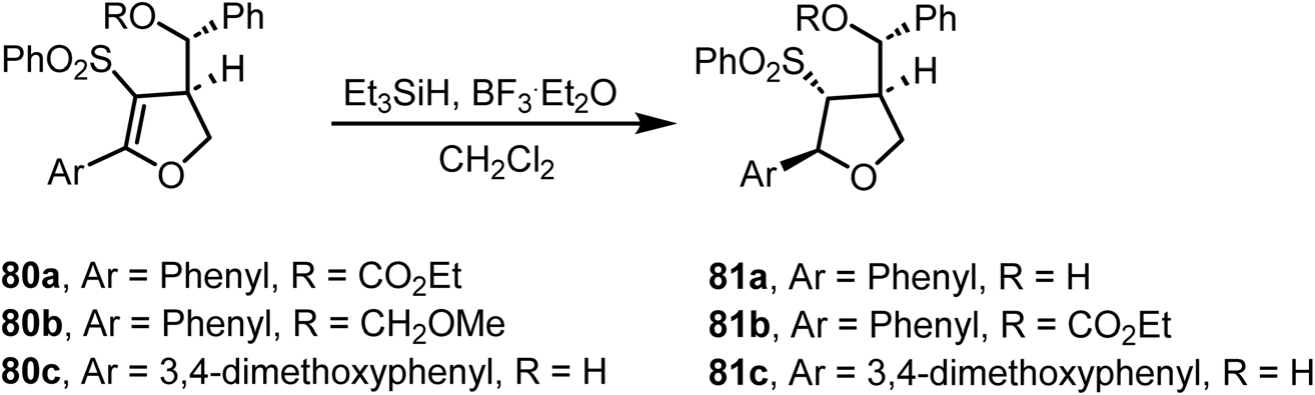
Reduction of dihydrofuran derivatives.

The stereoselective reduction^100^ of compounds 80 using ionic hydrogenation with Et_3_SiH and BF_3_·Et_2_O resulted in the corresponding tetrahydrofuran derivatives (Scheme 32). Reduction of the double bond in 80b was readily achieved by treating with Et_3_SiH and BF_3_·Et_2_O in dichloromethane, obtaining the alcohol 81a in 83% yield. Similarly, the tetrahydrofurans 81b and 81c were synthesized in 75% and 85% yields, respectively, from 80a and 80c (Scheme 32).

**Scheme 33:**
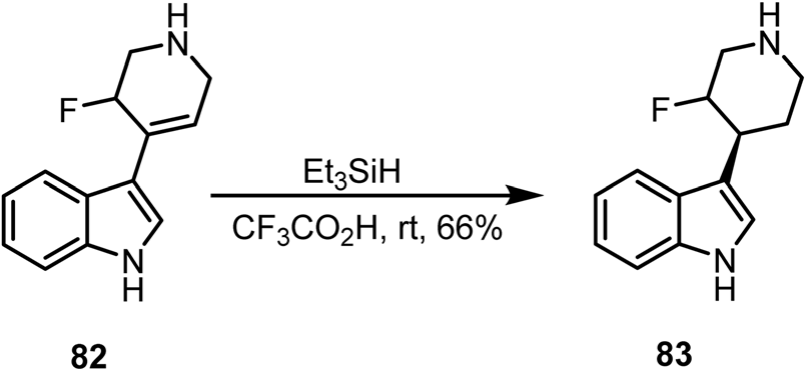
Reduction of a tetrahydropyridine by ionic hydrogenation.

The substitution at the C-3 position of the indole nucleus with certain double-bonded compounds can be selectively reduced by ionic hydrogenation.^101^ The tri-substituted double bond in tetrahydropyridine 82 on ionic reduction with trifluoroacetic acid and triethylsilane afforded *trans*-fluoropiperidine 83 in 66% yield (Scheme 33).

**Scheme 34:**
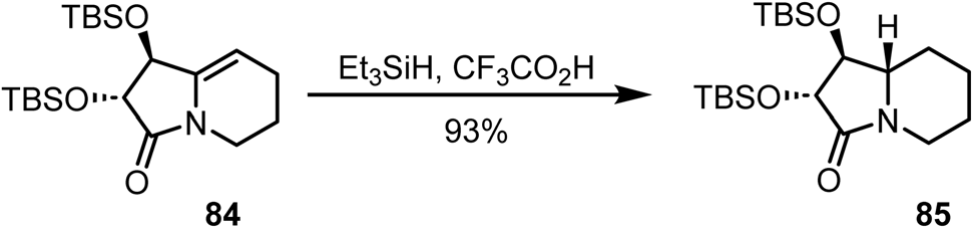
Stereoselective reduction of unsaturated lactam.

Ha and co-workers reported^102^ the stereoselective reduction of unsaturated lactam 84 with Et_3_SiH and CF_3_CO_2_H, which provided the saturated lactam 85 in 93% yield, serving as an intermediate in the synthesis of alkaloid (+)-lentiginosine (Scheme 34).

**Scheme 35:**
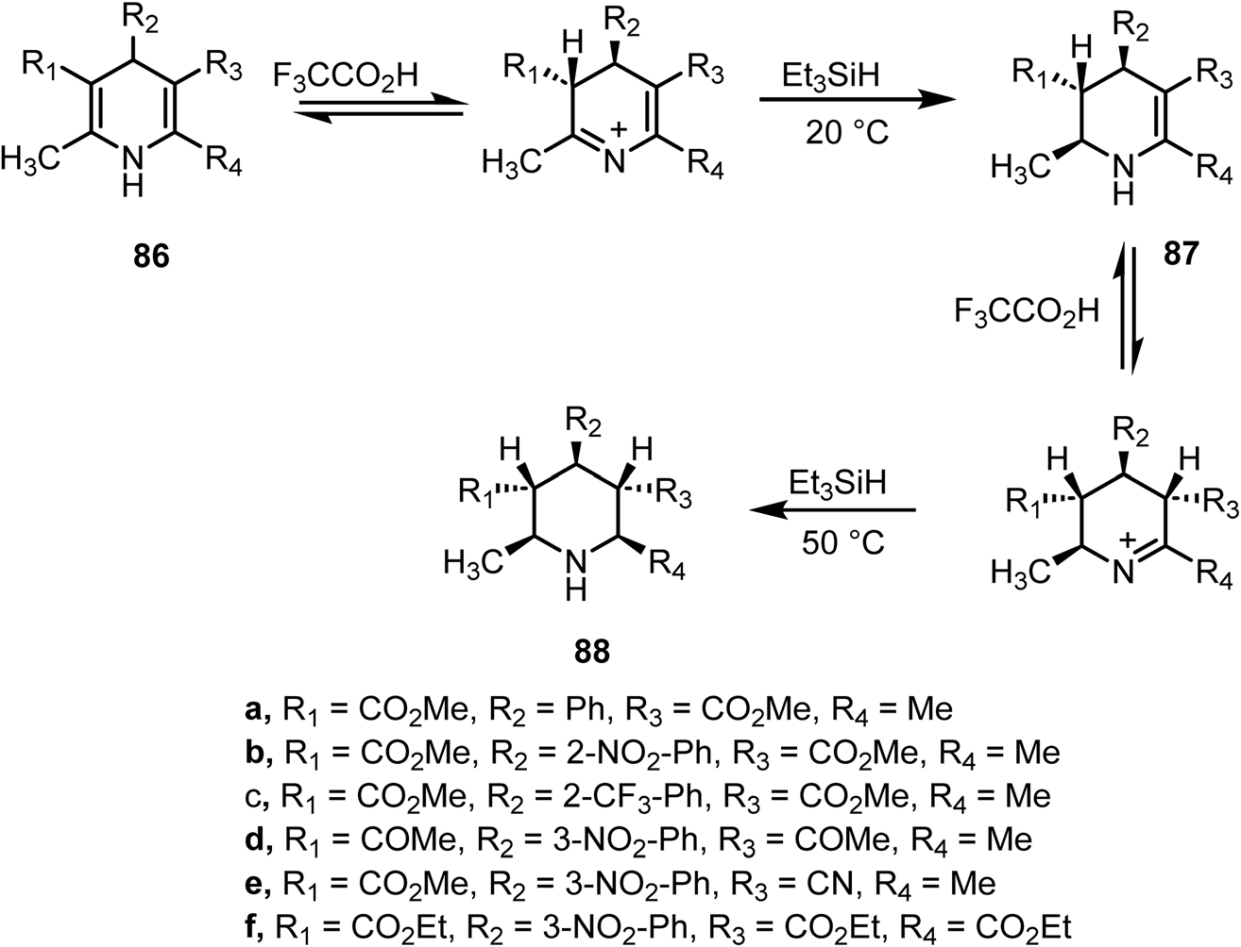
Ionic hydrogenation of 1,4-dihydropyridine derivatives.

Rosentreter reported^103^ the ionic hydrogenation of substituted 1,4-dihydropyridine 86 using triethylsilane and trifluoroacetic acid. With 1 equivalent of triethylsilane at room temperature, the partially reduced pyridine 87 was obtained selectively. Furthermore, the use of 3 equiv. of triethylsilane at 50 °C produced the corresponding piperidine derivatives 88 (Scheme 35).

**Scheme 36:**
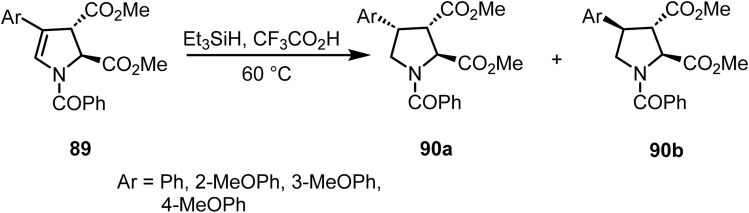
Ionic hydrogenation of dihydropyrrole derivatives.

Baldwin and co-workers demonstrated^104,105^ the synthesis of acromelic acid analogues *via* ionic hydrogenation of substituted dihydropyrrole derivatives 89 using triethylsilane in trifluoroacetic acid at 60 °C. This reaction resulted in epimers of the protected acromelic acid analogues 90a and 90b (1 : 1 ratio) in satisfactory yields (Scheme 36).

**Scheme 37:**
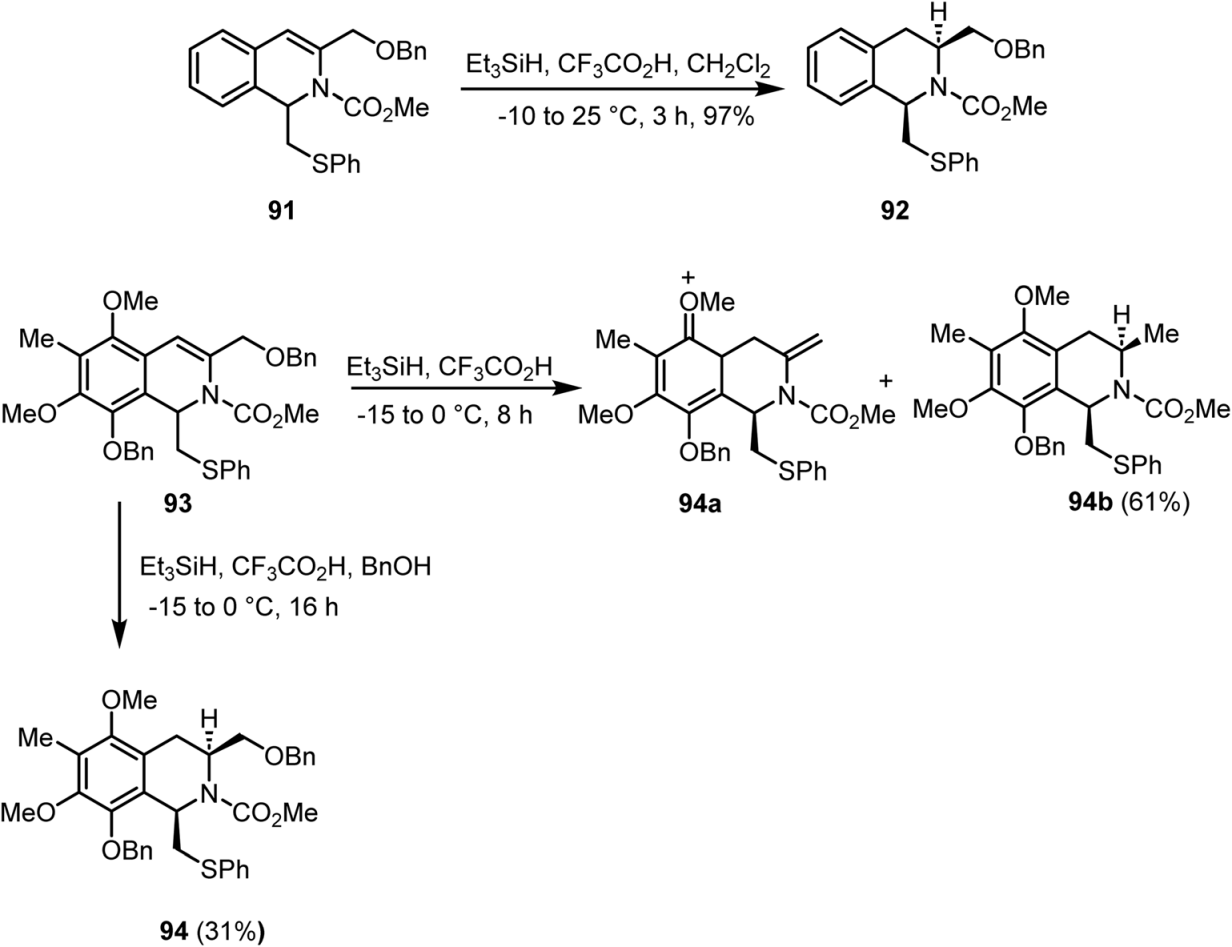
Ionic hydrogenation of double bonds in compounds 91 and 93.

Magnus and co-workers reported^106^ a synthetic strategy for the formation of 1,3-*cis*-substituted tetrahydroisoquinolines from *ortho*-iodo imines *via* Larock isoquinoline synthesis, organolithium addition to unactivated isoquinolines, and ionic hydrogenation. Compound 91, on reaction with CF_3_CO_2_H and triethylsilane in CH_2_Cl_2_ at −10 °C to 25 °C, afforded compound 92 in 97% yield (Scheme 36). Furthermore, reduction of the ene-carbamate moiety in 93 using Et_3_SiH and CF_3_CO_2_H in CH_2_Cl_2_ led to the competitive formation of 94b in 61% yield and the expected product 94 in 31% yield (Scheme 37). Moreover, performing the same reaction in the presence of benzyl alcohol (15 equiv.) enhanced the yield of 94 to 71%, while 94b was obtained in 22% yield.

**Scheme 38:**
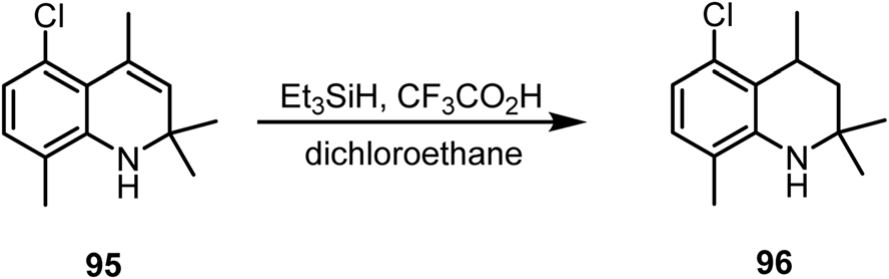
Ionic hydrogenation of dihydroquinoline.

Roach and co-workers reported^107^ the ionic hydrogenation of dihydroquinoline 95 with triethylsilane and trifluoroacetic acid in dichloroethane at 80 °C, yielding compound 96, as shown in Scheme 38.

**Scheme 39:**
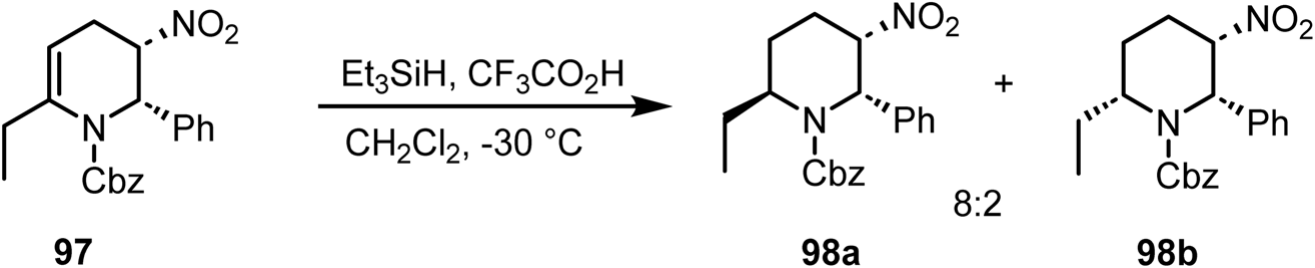
Regioselective reduction of tetrahydropyridine derivatives.

Humphrey and co-workers reported^108^ the regioselective reduction of the double bond in 95 with triethylsilane and trifluoroacetic acid in dichloromethane at −30 °C, which resulted in an 8 : 2 *trans*/*cis* ratio of 98a and 98b (Scheme 39). Compound 98a was obtained in 72% yield after crystallization. Moreover, the reaction conditions do not affect the reducible functionalities, NO_2_ and Cbz, present in compound 97.

**Scheme 40:**
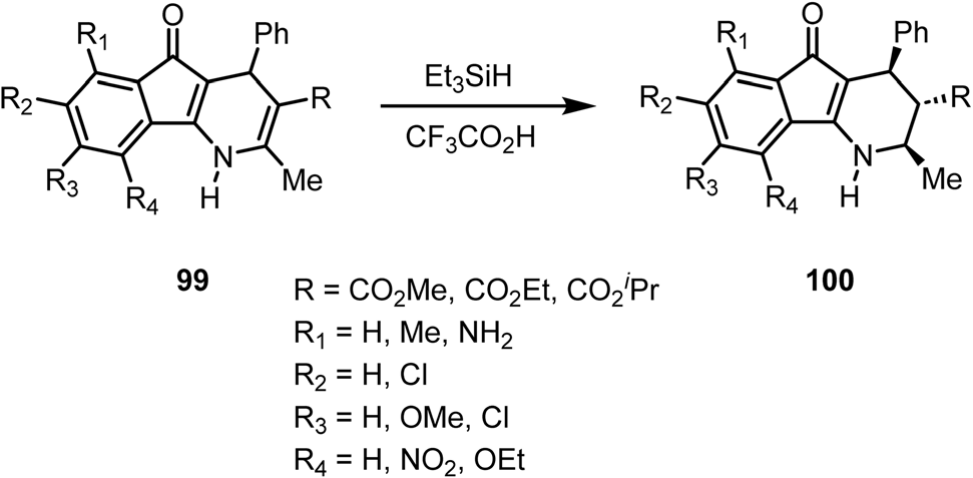
Regio- and chemo-selective reduction of dihydropyridine derivatives.

Stupnikova and co-workers reported^109^ the reduction of 5-oxo-4-phenyl-1*H*-4,5-dihydroindeno[1,2-*b*]pyridines 99 with triethylsilane in trifluoroacetic acid, which afforded the corresponding 1,2,3,4-tetrahydroindeno[1,2-*b*]pyridines 100 with a *trans*-configuration, as shown in Scheme 40.

**Scheme 41:**
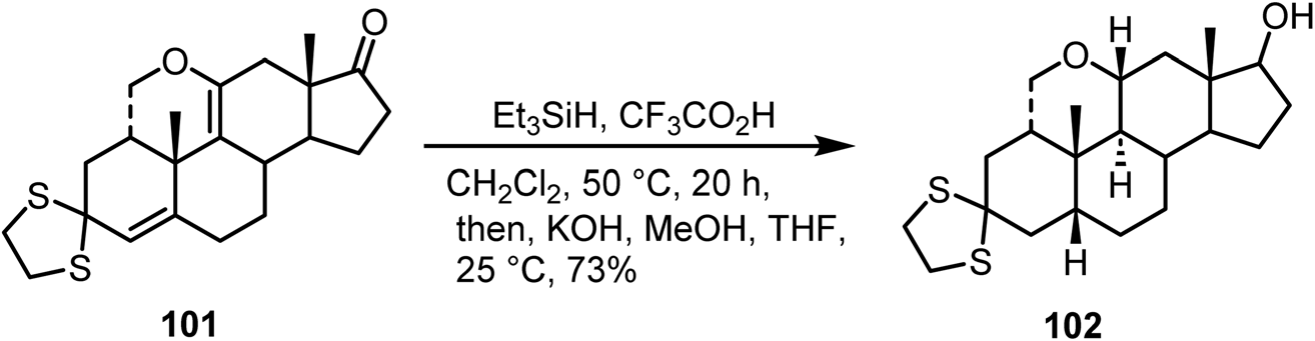
Reduction of double bonds and keto functionality.

Ibrahim-Quali reported^110^ the reduction of the Δ^9,11^ double bond in compound 101 using 10 equiv. of triethylsilane and 50 equiv. of trifluoroacetic acid, and after hydrolysis of the 17-trifluoroacetate group, the desired steroid analogue 102 was obtained in 73% yield (Scheme 41).

### 2.3 Reduction of cyclic double bonds in aromatic heterocycles

**Scheme 42:**
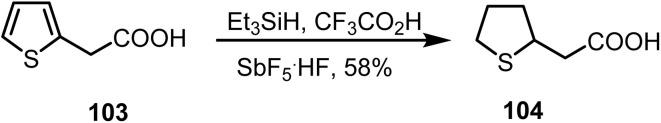
Ionic hydrogenation of thiophene.

The salient feature of ionic hydrogenation is that sulfur-containing compounds can be reduced, which typically poison catalysts in conventional hydrogenation methods. Using the silane-trifluoroacetic acid system, substrates such as thiophenes, benzothiophenes and octahydrothioxanthenes were successfully converted to their dihydro- and tetra-hydro derivatives. Thiophene-2-acetic acid 103 on ionic hydrogenation^111^ with Et_3_SiH in CF_3_CO_2_H containing a trace amount of superacid (HSbF_6_) afforded tetrahydro-thiophene-2-acetic acid 104 in 58% yield (Scheme 42).

**Scheme 43:**
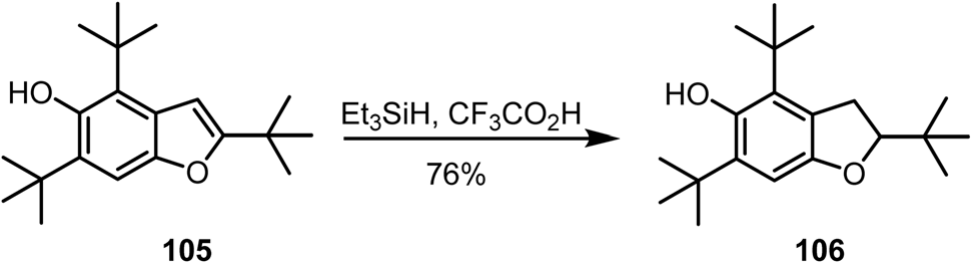
Ionic hydrogenation of a benzofuran derivative.

The highly substituted benzofuran derivative 105 on ionic hydrogenation^112^ with triethylsilane and trifluoroacetic acid from 0 °C to room temperature afforded the racemic dihydro-derivative 106 in 76% yield (Scheme 43).

**Scheme 44:**
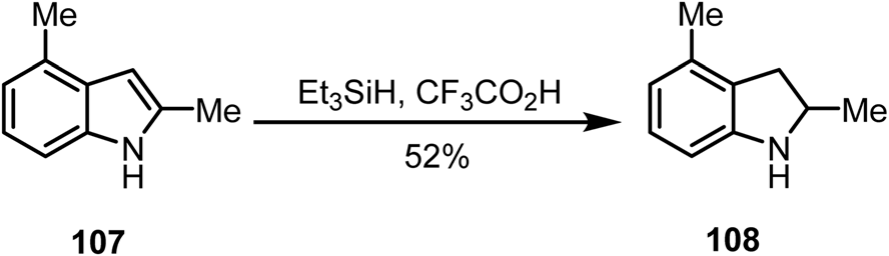
Reduction of indole derivative 107.

Electron-deficient aromatic heterocycles, such as pyridines and related compounds, are unreactive under ionic hydrogenation conditions. Therefore, this method is most suitable for the more reactive five-membered ring heteroaromatics. Moreover, indoles and pyrroles can be effectively reduced *via* ionic hydrogenation. Stachel and co-workers reported the ionic hydrogenation^113^ of 2,4-dimethyl indole 107 with triethylsilane in trifluoroacetic acid, which yielded 2,4-dimethyl-dihydroindole 108 in 52% yield (Scheme 44).

**Scheme 45:**
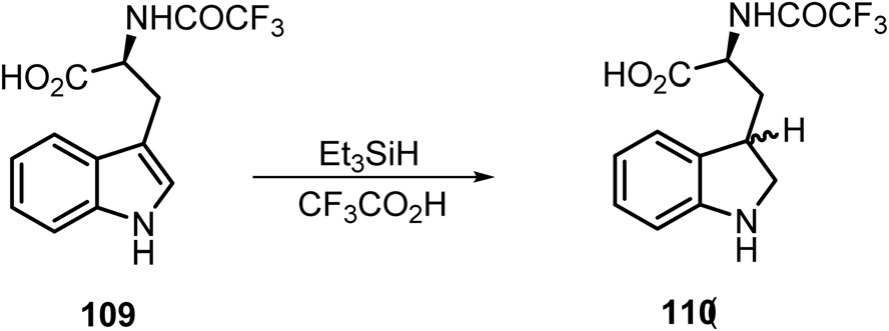
Reduction of indole derivative 109.

Carr and co-workers reported^114^ the ionic hydrogenation of the trifluoroacetyl derivative of l-tryptophan 109 at the C-2 double bond using CF_3_CO_2_H and Et_3_SiH. This led to a diastereomeric mixture of indolines 110 (45 : 55) in good yield (Scheme 45).

### 2.4 Miscellaneous

**Scheme 46:**
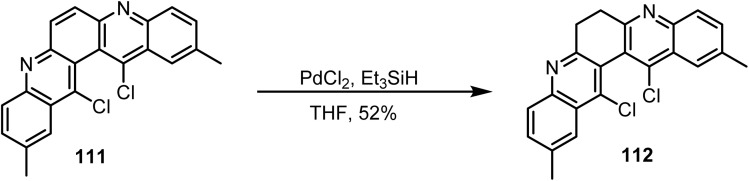
Selective reduction of the 6,7-double bond in compound 111.

Lartia and coworkers reported^115^ the selective reduction of the 5,7-double bond in compound 111 using triethylsilane and palladium chloride, which afforded the 6,7-dihydrogenated product 112 in 52% yield (Scheme 46).

**Scheme 47:**
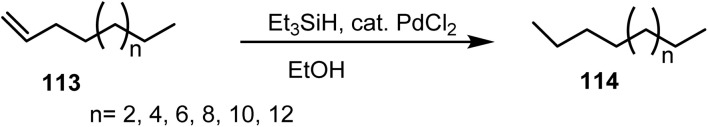
Reduction of 1-alkenes by triethylsilane and PdCl_2_.

Mirza-Aghayan and co-workers reported^116^ the reduction of 1-alkenes 113 using triethylsilane and palladium(ii) chloride in ethanol at room temperature, which afforded the corresponding alkanes 114 in excellent yields (Scheme 47).

Olah and co-workers reported^117^ the reduction of alkenes using triethylsilane, trifluoroacetic acid and ammonium fluoride in dichloromethane, which afforded the corresponding alkanes in good yields.

## 3. Summary

This review compiles a diverse and valuable collection of methodologies for the synthesis of fine chemicals, intermediates of complex molecules, natural products, and bioactive compounds. A wide range of alkene-containing substrates has been successfully reduced *via* ionic hydrogenation using triethylsilane and trifluoroacetic acid/Lewis acid, and related information is collected from the literature and described here. As demonstrated over the past four to five decades, continued advancement in this field holds promise for broader applications of ionic hydrogenation in synthetic organic chemistry. Future innovations will depend on a deeper mechanistic understanding and strategic application of the principles outlined in this review. We dedicate this work to the researchers who have contributed to the field of ionic hydrogenation and hope it serves to inspire the next generation of chemists to further expand its scope and utility.

## Conflicts of interest

The author declares that there is no financial or personal conflict of interest that could influence the integrity or outcomes of this study.

## Abbreviations

Ac: acetyl
Ar: aryl
aq.: aqueous
Bn: benzyl
Boc: *tert*-butyloxycarbonyl
°C: degree Celsius
cat.: catalytic
Cbz: benzyloxycarbonyl
dr: distereomeric ratio
equiv.: equivalent
Et: ethyl
h: hour (s)
MOM: methoxy methyl
Nap: naphthyl
Ph: phenyl
PMHS: polymethylhydrosiloxane
rt: room temperature
TBS: tertiary butyl dimethylsilyl
TES: triethylsilane
TFA: trifluoroacetic acid
THF: tetrahydrofuran
THP: tetrahydropyranyl
TMS: trimethylsilyl

## Data Availability

The information and data referenced in this review are derived from publicly accessible scientific publications, such as peer-reviewed journal articles. Proper attribution has been provided for all cited sources within the manuscript.
